# Transdifferentiating Astrocytes Into Neurons Using ASCL1 Functionalized With a Novel Intracellular Protein Delivery Technology

**DOI:** 10.3389/fbioe.2018.00173

**Published:** 2018-11-21

**Authors:** Meghan Robinson, Ian Fraser, Emily McKee, Kali Scheck, Lillian Chang, Stephanie M. Willerth

**Affiliations:** ^1^Division of Medical Sciences, University of Victoria, Victoria, BC, Canada; ^2^Biomedical Engineering Program, University of Victoria, Victoria, BC, Canada; ^3^Biology Program, University of Victoria, Victoria, BC, Canada; ^4^Biochemistry Program, Bates College, Lewiston, ME, United States; ^5^Mechanical Engineering, Faculty of Engineering, University of Victoria, Victoria, BC, Canada; ^6^Center for Biomedical Research, Faculty of Engineering, University of Victoria, Victoria, BC, Canada; ^7^International Collaboration for Repair Discovery, University of British Columbia, Vancouver, BC, Canada

**Keywords:** reprogramming, transcription factors, neuroscience, small molecules, drug delivery

## Abstract

Cellular transdifferentiation changes mature cells from one phenotype into another by altering their gene expression patterns. Manipulating expression of transcription factors, proteins that bind to DNA promoter regions, regulates the levels of key developmental genes. Viral delivery of transcription factors can efficiently reprogram somatic cells, but this method possesses undesirable side effects, including mutations leading to oncogenesis. Using protein transduction domains (PTDs) fused to transcription factors to deliver exogenous transcription factors serves as an alternative strategy that avoids the issues associated with DNA integration into the host genome. However, lysosomal degradation and inefficient nuclear localization pose significant barriers when performing PTD-mediated reprogramming. Here, we investigate a novel PTD by placing a secretion signal sequence next to a cleavage inhibition sequence at the end of the target transcription factor–achaete scute homolog 1 (ASCL1), a powerful regulator of neurogenesis, resulting in superior stability and nuclear localization. A fusion protein consisting of the amino acid sequence of ASCL1 transcription factor with this novel PTD added can transdifferentiate cerebral cortex astrocytes into neurons. Additionally, we show that the synergistic action of certain small molecules improves the efficiency of the transdifferentiation process. This study serves as the first step toward developing a clinically relevant *in vivo* transdifferentiation strategy for converting astrocytes into neurons.

## Introduction

Transdifferentiation converts mature cells from one specialized cell type into another by varying gene expression patterns (Tanabe et al. 2015). These expression patterns are controlled by transcription factors, regulatory proteins which bind to DNA promoter regions to activate transcription of key developmental genes. Ectopic expression of these transcription factors in somatic cells can change cell fates without the need for inducing a pluripotent state (Tanabe et al., 2015). This process avoids the risk of tumorigenesis associated with transplanting pluripotent stem cell-derived therapies *in vivo*, where incomplete differentiation leads to uncontrolled proliferation (Gordeeva and Khaydukov, 2017). The final genetic signatures of these transdifferentiated cells are stable and not restricted to the lineage of the original cell type, allowing cells to be sourced directly from endogenous tissue local to the site of injury (Tanabe et al., 2015). Performing *in vivo* transdifferentiation would eliminate the need for cell transplantation and immunosuppression depending on the target application.

Ectopic transcription factor expression can be efficiently accomplished through insertion into the genome by viral vectors encoding the amino acid sequence of the desired transcription factor. However, this method is not safe for *in vivo* use in clinical trials due to the risk of activating oncogenes by random DNA insertion into the host genome (Li et al., 2002; Modlich et al., 2006; Medvedev et al., 2010). Non-integrating vectors have been developed capable of transdifferentiation, but at lower efficiencies. Some well-known examples of non-integrating methods include vectors composed of single-stranded RNA, such as the Sendai virus, circular DNA, such as plasmid vectors and minicircles, and mRNA vectors, which reside in the cytoplasm where they are translated into proteins. Despite these improvements in non-viral delivery, vector-based transdifferentiation strategies still face challenges such as transgene silencing, inflammation due to the presence of residual bacterial DNA, and poor nuclear uptake due to their physical size (Hardee et al., 2017).

The search for non-integrating, non-viral reprogramming methods has led to the discovery of transdifferentiation using microRNAs and small molecules (Ma et al., 2017). In 2011, it was determined that the microRNAs miR-124 and miR-9/9^*^ were sufficient to induce neurons from fibroblasts (Ambasudhan et al., 2011; Yoo et al., 2011;). Later in 2014 Zhu et al. discovered that viral-mediated expression of octamer binding transcription factor 4 (OCT4) was sufficient to transdifferentiate somatic cells into neural stem cells when combined with small molecules that promote lineage-specific signaling (Zhu et al., 2014). Morphogens and bioactive small molecules can potentially enhance the action of transcription factors by improving chromatin accessibility, targeting key signaling pathways, and regulating metabolism (Lin and Wu, 2015). These results led several groups to identify combinations of small molecules capable of transdifferentiating somatic cells such as fibroblasts, astrocytes and even glioblastoma cells into neurons (Cheng et al., 2014; Hu et al., 2015; Li et al., 2015; Zhang et al., 2015, 2016; Gao et al., 2017; Lee et al., 2018). Although the transdifferentiation methods differ, these small molecule and microRNA protocols share a common thread in that they produce mainly one neuronal subtype–medium spiny neurons in the case of microRNAs, or interneurons in the small molecules methods with the exception of the glioblastoma-transdifferentiated neurons which resulted in immature post-mitotic neurons. The inability to produce the neuronal subtypes which are lost in neurodegenerative disorders like Parkinson's Disease, Alzheimer's Disease, amyotrophic lateral sclerosis (ALS), Huntingdon's Disease, spinal cord injury and blindness, represents a major limitation in current small molecule and microRNA reprogramming protocols.

Researchers have begun to explore protein transduction domains (PTDs), also known as cell penetrating peptides (CPPs), as tools for transdifferentiation to address these limitations (Zahid and Robbins, 2012). These CPPs are small peptides that penetrate the cell membrane to deliver bioactive macromolecules. The first PTD was derived from the transactivator of transcription (TAT) protein sequence of the human immunodeficiency virus (HIV), which binds to a phospholipid component of the inner surface of the cell membrane through a cationic poly-arginine sequence to enable cellular internalization (Yang et al., 2012b; Gordeeva and Khaydukov, 2017). Other examples of PTDs include Penetratin, a cationic arginine-rich sequence taken from the Drosophila protein antennapedia homeotic transcription factor (Antp), and CADY, a sequence combining arginine residues with aromatic tryptophan, whose helical conformation within the cellular membrane favors cellular uptake (Crombez et al., 2009; van den Berg and Dowdy, 2011). PTDs have the potential to be fitted with binding domains to target specific cell populations, making them ideal tools for *in vivo* reprogramming of endogenous cells (van den Berg and Dowdy, 2011; Zahid and Robbins, 2015). For example, C-end rule (CendR) is an amino acid sequence containing a carboxy (C)-terminal motif which binds to neuropilin-1, a cell surface receptor expressed in epithelial, neuronal, and cancer cells (Teesalu et al., 2009; Pang et al., 2014). In 2014, Hu et al. successfully transdifferentiated neuropilin-1 expressing retinal pigmented epithelial cells into neurons using a C-end rule PTD fused to the transcription factor self-determining region Y-box2 (SOX2) (Hu et al., 2014).

Little work has been done to develop such protocols to date despite the promise of PTDs as a tool for *in vivo* transdifferentiation. A search using the Pubmed database revealed only one further publication using PTD delivery where Islas et al. transdifferentiated human dermal fibroblasts into immature contractile cardiomyocytes (Islas et al., 2012). They fused TAT-PTDs to two cardiac transcription factors, erythroblastosis virus E26 oncogene homolog 2 (ETS2) and mesoderm posterior 1 (MESP1). Treatment with these TAT-PTDs followed by treatment with cardiac gene-inducing morphogens activin A and BMP2 resulted in successful transdifferentiation of fibroblasts into functional cardiomyocytes. Morphogen treatment significantly improved induction of key cardiac genes, whereas addition of the morphogens alone had no effect, further illustrating the transdifferentiation potential of combining morphogens or bioactive small molecules with transcription factors.

In this work, we developed a protocol using a novel PTD to transdifferentiate somatic cells into interneurons. We hypothesized that the delivery of the neural transcription factor achaete scute homolog 1 (ASCL1) by this novel PTD design in combination with the action of bioactive small molecules would promote neuronal transdifferentiation of astrocytes, the most abundant cell type in the central nervous system. ASCL1 is a transcription factor and master regulator of neurogenesis (Vasconcelos and Castro, 2014). Its ability to bind readily to closed chromatin and promote the appearance of new regions of open chromatin allows coordinated activation of previously silenced genes (Iwafuchi-Doi and Zaret, 2014; Raposo et al., 2015). In 2015, Liu et al. showed that *in vivo* viral delivery of ASCL1 to mouse dorsal midbrain astrocytes was sufficient for neural transdifferentiation, with 76.8% of astrocytes adopting a neural fate by day 10 (Liu et al., 2015). ASCL1 was also chosen for its versatility. While the action of ASCL1 alone leads to an interneuron fate, it is known to induce dopaminergic, cholinergic or serotonergic neuronal subtypes when combined with dopaminergic factors LMX1B/NURR1 (Addis et al., 2011; Theka et al., 2013), cholinergic factors MYT1L/BYRN2/LHX3/HB9/ISL1/NGN2 (Son et al., 2011), or serotonergic factors FOXA2/LMX1B/FEV (Xu et al., 2016). The PTD employed for these experiments was designed by iProgen Biotech Inc. to take advantage of the cell's innate retrograde transport pathway by integrating a secretion signal peptide and a cleavage inhibition peptide to a cargo protein (Lee et al., 2013). Initially the PTD integrates itself into the cell surface membrane due to its amphipathic nature. Once internalized, the secretion signal peptide preferentially binds to its native binding partner, translocon, a complex of proteins tasked with translocating polypeptides with a targeting signal sequence into the interior space of the endoplasmic reticulum (ER). As the translocon retrogrades back through the ER into the cytoplasm, so does the cargo. This combination of secretion signal peptide and cleavage inhibition peptide, termed IPTD, shows greatly improved delivery of proteins into the cytoplasmic space over conventional PTDs such as TAT and poly-arginine. In 2016, we showed that ASCL1-IPTD could efficiently generate neurons from human induced pluripotent stem cells (hiPSCs) (Robinson et al., 2016). We show here that ASCL1-IPTD in combination with the small molecules LDN193189, SB431542, DAPT and valproic acid can rapidly reprogram astrocytes into mature GABAergic and glutamatergic interneurons with high efficiency, and that this process is unique to the transcription factor ASCL1. We also show that in the absence of these small molecules ASCL1-IPTD cannot transdifferentiate into a neuronal fate, but does generate a divergent cell fate of myoblasts. Because of its inherent versatility in generating other neuronal subtypes, this ASCL1-IPTD protocol serves as a starting point for further development through the functionalization of subtype-specific factors with IPTD to generate subtypes of neurons. Additionally, the use of IPTD allows for future adaption with specific tissue-targeting motifs. This method can be applied as novel treatment for neurodegenerative diseases or spinal cord injury by transdifferentiating astrocytes into specific neuronal populations as a way to restore function.

## Materials and methods

### Cell culture

Human fetal astrocytes (ScienCell cat#1800) were cultured on poly-L-lysine coated plates in Astrocyte Media (ScienCell). Media was changed every 2 days and cells were passaged 1:6 when 90% confluent using trypsin-EDTA (ScienCell). 10% fetal bovine serum (FBS, Gibco) was added to media for 6 passages (6 weeks) as previously described to purify astrocyte cultures of neural progenitors (Zhang et al., 2015). Astrocytes were incubated for 24 h on an orbital shaker plate at 200 rpm as previously described to purify low passage (P2) astrocytes of neural progenitors (Lian et al., 2016). Mouse embryonic fibroblasts (MEFs) were purchased from MTI GlobalStem and thawed onto poly-L-ornithine (PLO)/laminin (Sigma Aldrich) substrates in high glucose DMEM (Gibco) supplemented with 10% FBS.

### Astrocyte reprogramming

Astrocytes were grown to confluence before reprogramming. Cells were cultured on poly-L-lysine (PLO)/laminin substrates and media was changed to high glucose Dulbecco's Modified Eagle Medium (DMEM) with 2 mM Glutamax (Gibco), 1% N2/B27 (ThermoFisher Scientific), 1% Human Serum Replacement 3 (Sigma Aldrich), and 1% penicillin/streptomycin (ThermoFisher Scientific) for cellular reprogramming experiments. 15 μg/mL ASCL-IPTD (iProgen Biotech) was supplemented for all 12 days, with 50% media changes every 2 days. For days 1-2, 5μM SB431542 (Stemcell Technologies) and 0.25 μM LDN193189 (Stemcell Technologies) were added to cultures. For days 3–12 the following small molecules were added: forskolin (F, 10 μM, Medchem Express), CHIR99021 (C, 1.5 μM, Medchem Express), ISX9 (I, 20 μM, Medchem Express), DAPT (D, 5 μM, Medchem Express). The combinations tested were C, F, D, I, CF, CI, CD, FI, FD, ID, FCI, FCD, CID, FID, CFID. For days 3–6, 0.5 μM valproic acid (Stemcell Technologies) was also added to improve chromatin accessibility.

### Fibroblast reprogramming

MEFs were seeded 40,000 cells/cm^2^ on PLO/laminin and cultured for 24 h in high glucose DMEM + 10% FBS to adhere. Media was then switched to high glucose DMEM, 1% N2/B27, 1% Human Serum Replacement 3, 2 mM GlutaMAX, 1% penicillin/streptomycin, 15 μg/mL ASCL1-IPTD and 20 mM forskolin for 14 days, with media changes of 50% every 2nd day.

### Immunocytochemistry

Immunocytochemistry was performed as described previously (Agbay et al., 2018). Cells were fixed with 3.7% formaldehyde/phosphate buffered saline (PBS) solution for 1 h, then permeabilized in 0.1% Triton X-100 (Sigma Aldrich) in PBS for 45 min at 4°C, then blocked with 5% normal goat serum (NGS, Sigma Aldrich) in PBS for 2 h at 4°C. Cells were then incubated with the primary antibodies mouse anti-TUJ1 (Millipore), rabbit anti-MYH3 (Abcam), rabbit anti-ASCL1 (Abcam), rabbit anti-DCX (Abcam), rabbit anti-SOX2 (Abcam), rabbit anti-MAP2 (Abcam), rabbit anti-GLUT1 (Abcam), rabbit anti-GAD65/67 (Abcam), mouse anti-NEUN (Novus Biologicals), mouse anti-TH (Stemcell Technologies), or goat anti-CHAT (Millipore), diluted 1:500 in PBS, and incubated overnight at 4°C in the dark. The next day cells were washed with PBS three times for 15 min each, and incubated with secondary antibodies goat anti-mouse IgG AlexaFluor488 (ThermoFisher Scientific), goat anti-rabbit IgG AlexaFluor568 (ThermoFisher Scientific), or donkey anti-goat AlexaFluor405 (Abcam) diluted 1:200 in PBS for 4 h at 4°C. Cells were washed again three times as previously described. For cells counterstained with DAPI nucleic acid stain, a 300 nM DAPI solution (Invitrogen) in PBS was added to the cultures after the final wash and incubated for 3 min, followed by rinsing with PBS. Cells were visualized and imaged with a Cytation5 (Biotek) Imaging platform at 20 × magnification. Fluorescently labeled cells were visualized with Biotek Dapi (377/477), GFP (469/525), and Texas Red (586/647) filter cubes, using dichroic mirror-based wavelength selection.

### Flow cytometry

Flow cytometry was performed as previously described (Montgomery et al., 2015). The cells were stained for SOX2-PE Mouse IgG2A (R&D Systems) with IgG2A-PE isotype control (R&D Systems), TUJ1-PerCP Mouse IgG2A (R&D Systems) with IgG2A-PerCP isotype control (R&D Systems), MAP2-PE Mouse IgG1 (Clone AP20, Milli-Mark) with Mouse IgG1 PE isotype control (R&D Systems), and NEUN-PE Mouse IgG1 (Clone A60) with Mouse IgG1 PE isotype control (R&D Systems). Cells were collected rinsed with PBS 3 times, followed by incubation in fixation/permeabilization buffer (R&D Systems) for 10 min, and 3 more washes with PBS. Next cells were resuspended in permeabalization/wash buffer (R&D Systems) with each antibody, diluted as per manufacturer's instructions, for 45 min at 4°C in the dark. Cells were then washed in permeabilization/wash buffer 3 times. Data was collected using the Millipore Guava EasyCyte HT flow cytometer. Cells were gated to exclude small debris and clustered cells, and a maximum of 5,000 gated events were collected for each sample. All analysis was completed using GuavaSoft EasyCyte software.

### Quantification of transdifferentiated neurons

Cells were first immunostained with the neuronal cytoskeletal marker TUJ1 to quantify transdifferentiated neurons in each group of the small molecule screen. Next, a grid of 5 × 5 images was taken in each of 3 wells for each group (25 images per well, *n* = 3 biological replicates). Cells were visualized with a Leica DMI 3000B microscope equipped with an XCite Series 120Q fluorescent light source and QImaging RETIGA 2000R camera at 100 × magnification, and images were captured using QCapture Software 2.9.12 with a GFP filter (blue/470, Leica Systems) and a DAPI filter (400, Leica Systems) and dichroic mirrors (500, Leica Systems). Cells were counted manually if they were positive for TUJ1 and possessed a small rounded soma, with at least 2 neuritic processes. Total cells were counted by DAPI stain using ImageJ: images were made binary and a watershed was applied to separate nuclei that were touching by a single pixel width, and each cell was counted as a particle under the Analyze-Particles Menu.

### Statistical analysis

Statistical analysis was performed by a one-way ANOVA followed by a one-tailed Student's *t*-test with a 95% confidence level (α = 0.05). Results are presented as the mean ± standard deviation. All experiments were carried out with a biological *n* = 3.

## Results

### Transdifferentiation protocol developmental

**Figure 1:**
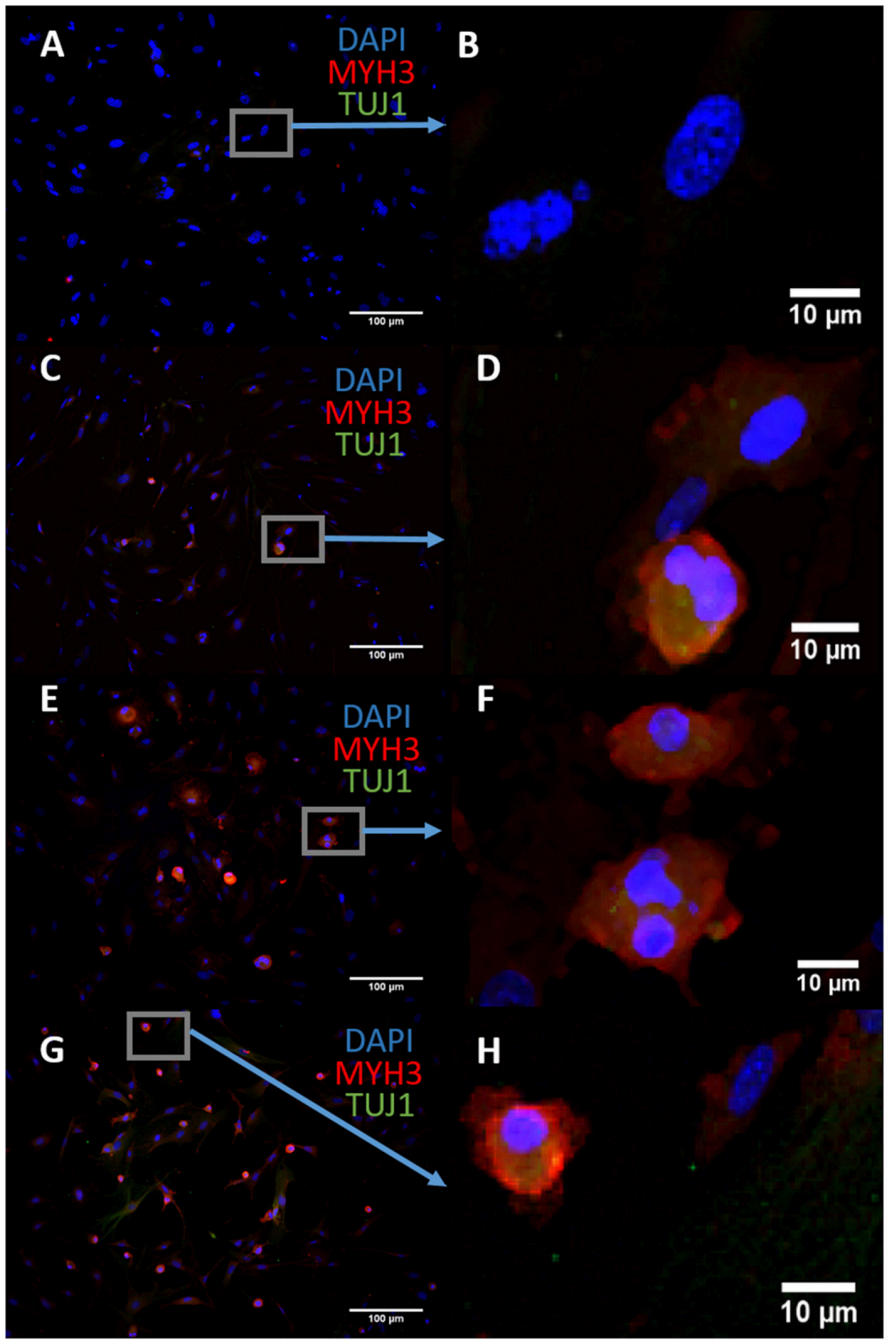
Mouse embryonic fibroblasts (MEFs) express the skeletal myoblast gene myosin heavy chain 3 (MYH3) and lack the neuronal gene beta tubulin (TUJ1) after 14 days of exposure to ASCL1-IPTD and the cAMP activator forskolin, indicating that they have adopted a myogenic cell fate rather than a neuronal cell fate. **(A)** Control. Scale bar is 100 μm. **(B)** High magnification of **(A)**. Scale bar is 10 μm. **(C)** Forskolin only. Scale bar is 100 μm. **(D)** High magnification of **(C)**. Scale bar is 10 μm. **(E)** ASCL1-IPTD only. Scale bar is 100 μm. **(F)** High magnification of **(E)**. Scale bar is 10 μm. **(G)** ASCL1-IPTD with forskolin. Scale bar is 100 μm. **(H)** High magnification of **(H)**. Scale bar is 10 μm.

We sought to characterize the transdifferentiation strength of ASCL1-IPTD before designing our protocol. We executed a well-known protocol where ectopic expression of ASCL1 alone converts mouse embryonic fibroblasts (MEFs) into neurons, and at lower expression levels, into skeletal myoblasts (Treutlein et al., 2016). The addition of the small molecule forskolin, an activator of cyclic adenosine monophosphate (cAMP) signaling, dramatically improves the efficiency of this protocol and so it was tested in parallel to determine if conversion efficiency could be similarly improved with ASCL1-IPTD (Shi et al., 2016). After 14 days of treatment, skeletal myoblasts were generated instead of neurons as indicated by myosin heavy chain 3 (MYH3) expression, with or without the addition of forskolin (Figure 1). Increasing the concentration of ASCL1-IPTD 5-fold also had no effect in generating neurons (Supplemental Figure 1).

**Figure 2:**
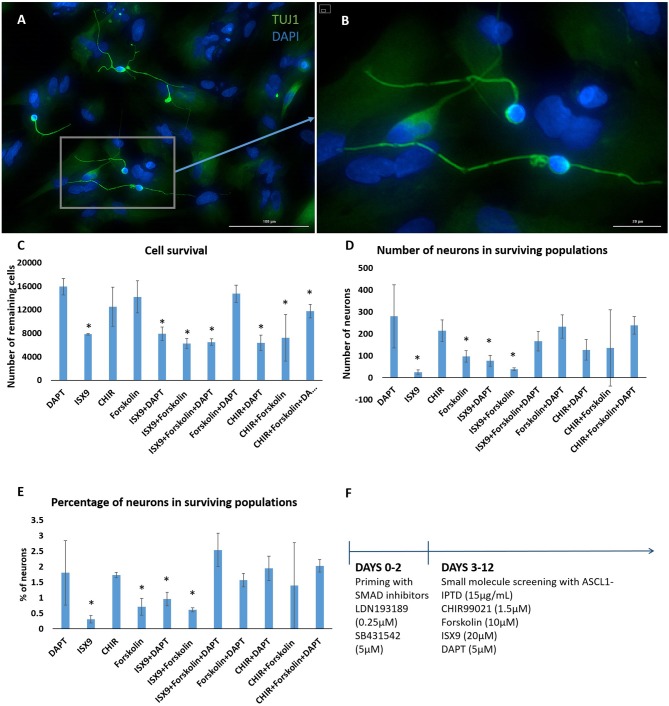
Small molecule screening of combinations of forskolin, ISX9, DAPT and CHIR99021 with ASCL1-IPTD show that DAPT is the most potent inducer of neural transdifferentiation. **(A)** Cells express beta tubulin (TUJ1), an early neuronal marker, and ASCL1 after 2 days of priming with LDN193189 and SB431542 and ASCL1-IPTD followed by 10 days of treatment with ASCL1-IPTD and DAPT. Scale bar is 100 μm. **(B)** High magnification of **(A)**. Scale bar is 20 μm. **(C)** Cell survival varied in each condition, with the DAPT alone condition being the least toxic by far (*n* = 3). **(D)** Neurons counted from each small molecule combination on day 12 (*n* = 3). **(E)** Neuronal conversion percentages. **(F)** Protocol timeline: 2 days of priming with SMAD inhibitors LDN193189 and SB431542 followed by 10 days of small molecule combinations. All 12 days included the addition of ASCL1-IPTD. Statistical analysis was performed by a one-way ANOVA followed by a one-tailed Student's *t*-test with a 95% confidence level (α = 0.05). Results are presented as the mean ± standard deviation, with a ^*^ representing a statistically significant difference between that condition and the DAPT alone condition.

We concluded that the strength of ASCL1-IPTD was not meeting the threshold expression level necessary for neuronal transdifferentiation. Accordingly, we adopted the strategy to first lower this threshold by repressing astrocytic gene expression using small molecules to inhibit transforming growth factor β1 (TGF-β1)/SMAD and bone morphogenic protein (BMP)/SMAD signaling pathways, which are jointly responsible for astrocyte commitment and maintenance (Stipursky and Gomes, 2007; Chambers et al., 2009; Yang et al., 2013; Zhang et al., 2015). Human cerebral cortex astrocytes were primed for 2 days followed by screening of a set of small molecules known to have neuronal transdifferentiation potential: CHIR99021, an inhibitor of glycogen synthase kinase 3 (GSK3) signaling; DAPT, an inhibitor of Notch signaling; forskolin, an activator of cAMP signaling; and isoxazole 9 (ISX9), an activator of calcium-mediated signaling (Zhang et al., 2015; Gascón et al., 2016, 2017; Gao et al., 2017). For days 3–6, valproic acid, a histone deacetylase inhibitor, was added to enhance chromatin accessibility (Huangfu et al., 2008). ISX9 induced a significant degree of toxicity in cultures, and the combination of CHIR99021 and ISX9 caused all the cells to detach from the plate (data not shown). All remaining combinations produced some degree of transdifferentiation from astrocytes into neurons within 10 days, with DAPT alone being the most effective enhancer of this process (Figure 2).

### The action of DAPT does not translate to NGN2-mediated transdifferentiation

**Figure 3:**
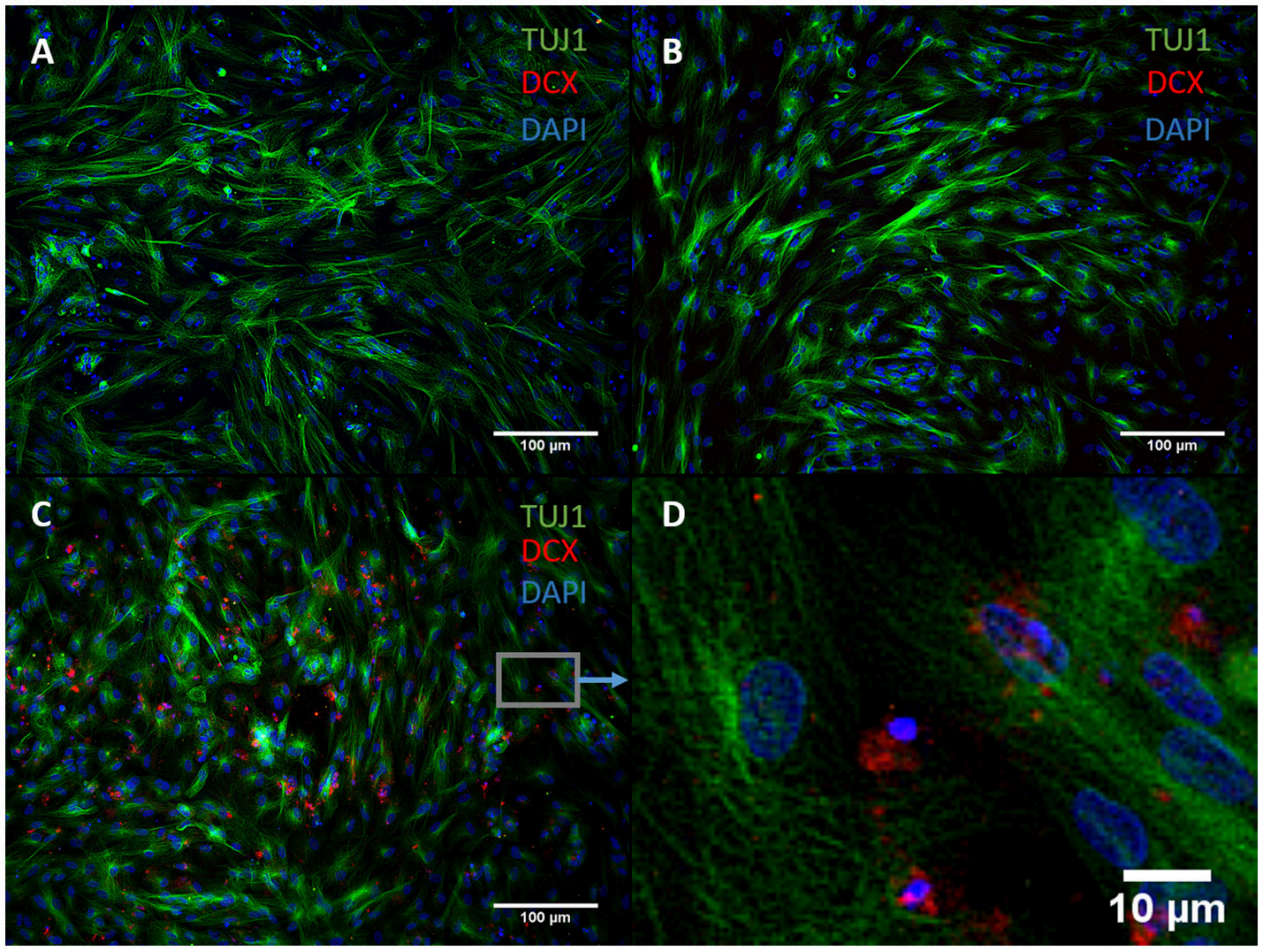
NGN2-IPTD in place of ASCL1-IPTD does not generate neurons, but does produce cells expressing doublecortin (DCX), a marker for neuroblasts. **(A)** Control. Scale bar is 100 μm. **(B)** Small molecules only. Scale bar is 100 μm. **(C)** NGN2-IPTD plus small molecules. Scale bar is 100 μm. **(D)** High magnification image of **(C)**. Scale bar is 10 μm. Note that TUJ1 is weakly expressed in astrocytes in addition to neurons and can be regarded as background stain in these images.

We exchanged ASCL1-IPTD with another functionalized neurogenic transcription factor, NGN2-IPTD to determine if ASCL1 could be replaced with NGN2. NGN2 also serves as a master regulator of neural fate, and when delivered virally is sufficient to transdifferentiate astrocytes into neurons with high efficiency (71%) (Parras et al., 2002; Berninger et al., 2007). However, replacing ASCL1-IPTD with NGN2-IPTD in combination with DAPT did not generate neurons, but was seen by immunocytochemistry to generate a population of cells expressing the neuroblast gene doublecortin (DCX), suggesting that ASCL1 and NGN2 are not interchangeable in our protocol (Figure 3).

### Astrocytic plasticity dramatically improves transdifferentiation of neurons

**Figure 4:**
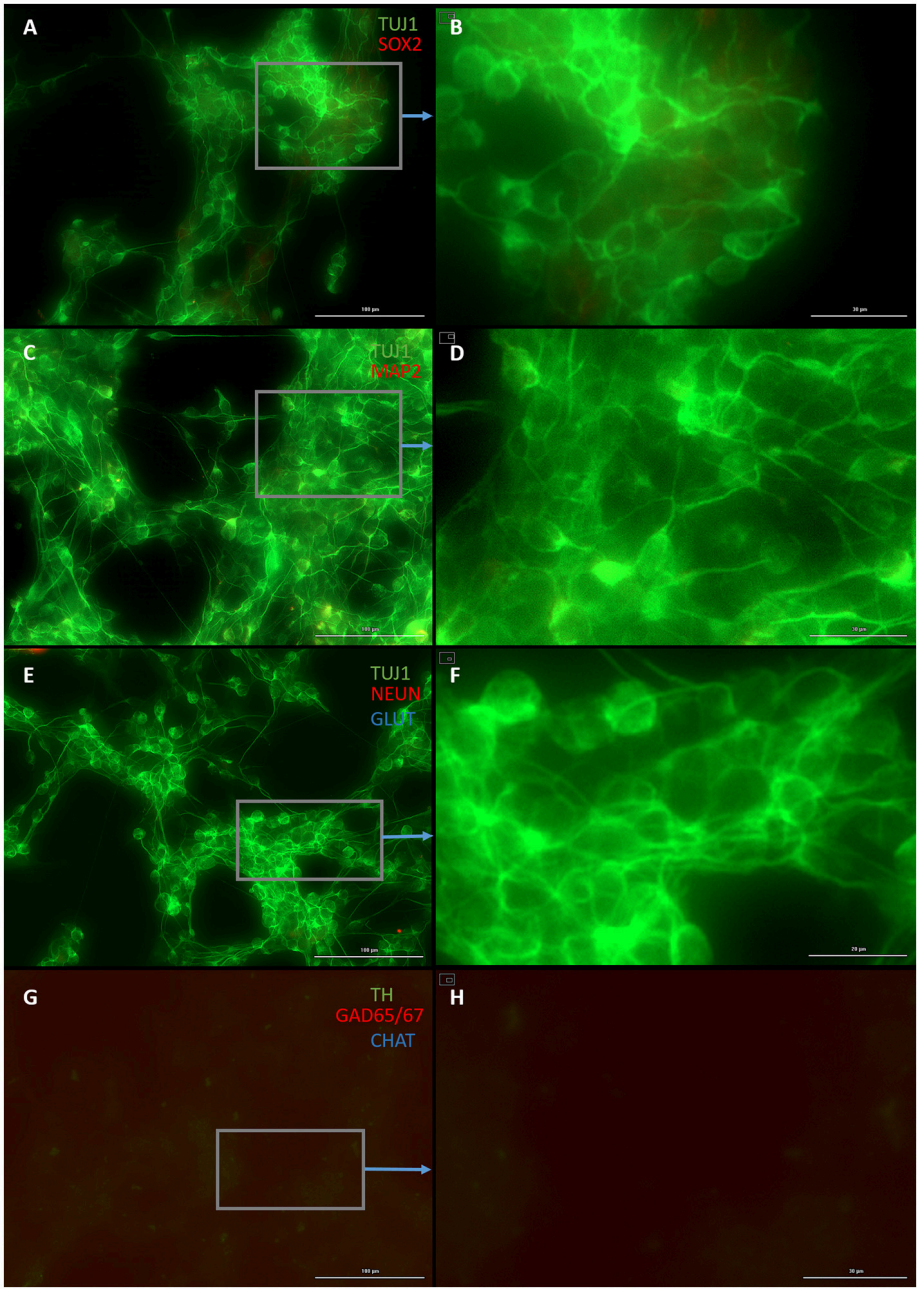
Small molecule only group after 2 days of priming by LDN193189 and SB431542 followed by 10 days of treatment with DAPT. **(A)** Cells express the early neural marker TUJ1 and weakly express the neural stem cell marker SOX2. Scale bar is 100 μm. **(B)** High magnification of **(A)**. Scale bar is 30 μm. **(C)** Cells are negative for the mature neural marker MAP2. Scale bar is 100 μm. **(D)** High magnification of **(C)**. Scale bar is 10 μm **(E)** Cells are negative for the mature neural marker NEUN and the neurotransmitter glutamate expressed by excitatory interneurons GLUT. Scale bar is 100 μm. **(F)** High magnification of **(E)**. Scale bar is 20 μm. **(G)** Cells are negative for the neurotransmitter tyrosine hydroxylase expressed by dopaminergic neurons TH, the neurotransmitter glutamic acid decarboxylase expressed by inhibitory interneurons GAD65/67, and the neurotransmitter choline acetyltransferase expressed by motor neurons CHAT. Scale bar is 100 μm. **(H)** High magnification of **(G)**. Scale bar is 30 μm. Note that some fluorescence is picked up by all the cells producing background fluorescence which can be ignored.

**Figure 5:**
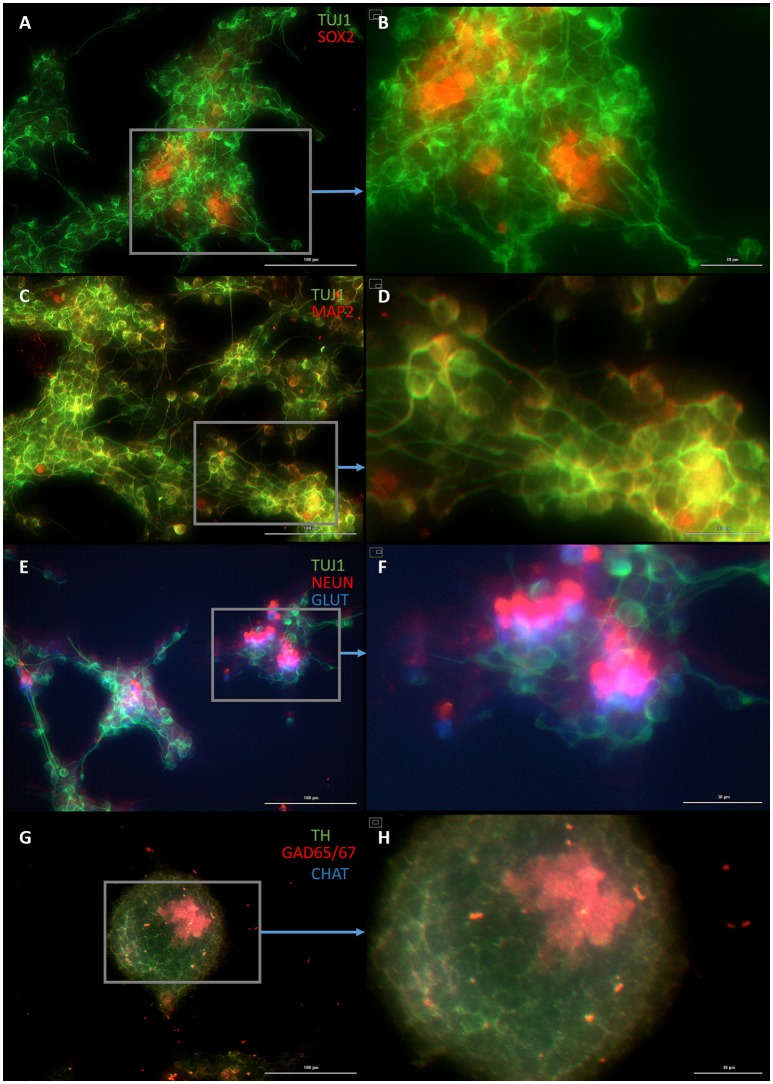
Plastic astrocytes generate neurospheres and mature neurons after 12 days of exposure to ASCL1-IPTD, including 2 days of priming by LDN193189 and SB431542 followed by 10 days of DAPT. **(A)** Cells express the early neural marker TUJ1 and the neural stem cell marker SOX2. Scale bar is 100 μm. **(B)** High magnification of **(A)**. Scale bar is 30 μm. **(C)** Cells express the mature neural marker MAP2. Scale bar is 100 μm. **(D)** High magnification of **(C)**. Scale bar is 30 μm **(E)** Cells express the mature neural marker NEUN and the neurotransmitter glutamate expressed by excitatory interneurons GLUT. Scale bar is 100 μm. **(F)** High magnification of **(E)**. Scale bar is 30 μm. **(G)** Cells are negative for the neurotransmitter tyrosine hydroxylase expressed by dopaminergic neurons TH, and negative for the neurotransmitter choline acetyltransferase expressed by motor neurons CHAT, but do express the neurotransmitter glutamic acid decarboxylase expressed by inhibitory interneurons GAD65/67. Scale bar is 100 μm. **(H)** High magnification of **(G)**. Scale bar is 30μm. Note that some fluorescence is picked up by all the cells producing background fluorescence which can be ignored.

**Figure 6:**
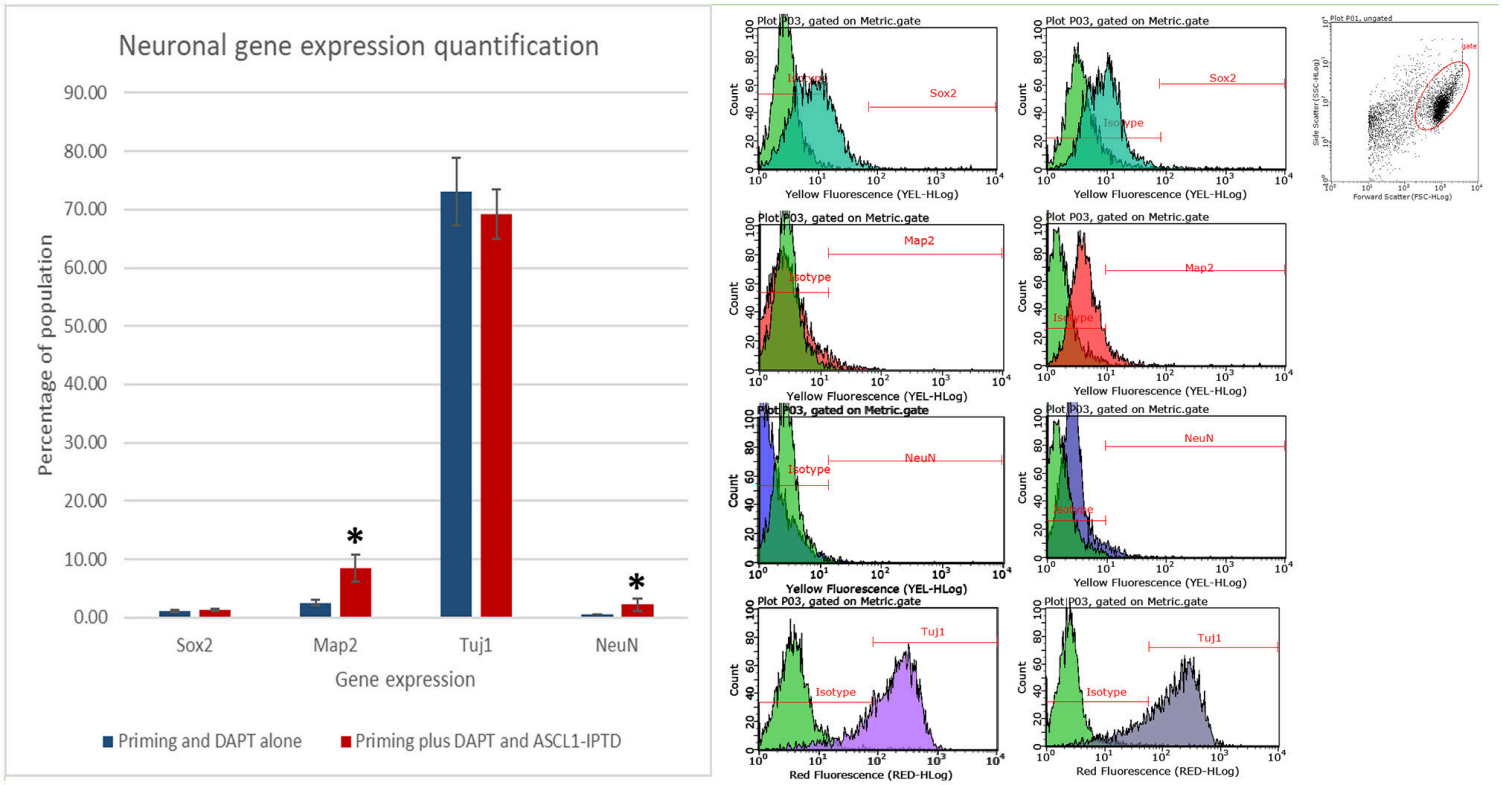
Quantification of neural gene expression for the immature neural marker TUJ1 and the mature neuronal markers MAP2 and NEUN by flow cytometry. Plastic astrocytes were exposed to 2 days of priming by LDN193189 and SB431542 followed by 10 days of exposure to DAPT, with or without ASCL1-IPTD. Results show that TUJ1 expression is similar in both groups, whereas MAP2 and NEUN expression are significantly upregulated in the ASCL1-IPTD group. Statistical analysis was performed by a one-way ANOVA followed by a one-tailed Student's t-test with a 95% confidence level (α = 0.05). Results are presented as the mean ± standard deviation, with a ^*^ representing a statistically significant difference between the ASCL1-IPTD group with the DAPT Alone group.

Both early astrocytes and astrocytes found at the site of injury possess the ability to form multi-potent neurospheres, making them highly plastic (Yang et al., 2012a; Götz et al., 2015; Sirko et al., 2015; Wang et al., 2015; Michelucci et al., 2016). We investigated the extent to which our protocol might transdifferentiate plastic astrocytes by applying it to early passage (P3) astrocytes. Indeed, analysis by flow cytometry showed that efficiency was dramatically improved to 73.09 ± 5.83% (from 1.81 ± 1.04%) by priming and DAPT exposure alone (Figures 2G,4,6), while the further addition of ASCL-IPTD resulted in abundant neurosphere formation, giving rise to cells expressing the mature neuronal markers MAP2 (8.48 ± 2.29%, vs. 2.56 ± 0.47% without) and NEUN (2.20 ± 1.01% with ASCL1-IPTD vs. 0.47 ± 0.05% without) by day 12 (Figures 5, 6). These neurons were seen by immunocytochemistry to express both glutamatergic and GABAergic neurotransmitter genes, suggesting a mix of excitatory and inhibitory interneurons, while treatment with small molecules alone were negative for neurotransmitter markers (Figure 5).

## Discussion

In this work, we show that ASCL1-IPTD combined with the complementary action of SMAD and Notch signaling inhibition serves an efficient inducer of neurons from astrocytes, particularly those which exhibit plasticity. This effect is important since astrocytes become reactive at the site of injury *in vivo* and acquire enhanced plasticity, enabling them to proliferate and de-differentiate to generate multipotent neurospheres with the potential to self-renew and give rise to astrocytes, neurons or oligodendrocytes (Yang et al., 2012a; Götz et al., 2015; Sirko et al., 2015; Wang et al., 2015; Michelucci et al., 2016). SMAD signaling regulates genes involved in astrocyte commitment and maintenance (Stipursky and Gomes, 2007; Chambers et al., 2009; Yang et al., 2013; Zhang et al., 2015), therefore we treated cells with SMAD inhibitors from days 1 to 2 to disrupt astrocyte commitment. Our screening process identified DAPT to be the most potent enhancer of ASCL1-IPTD in transdifferentiating neurons from astrocytes. Notch signaling maintains neural progenitors in a state of proliferation by repressing genes responsible for cell cycle arrest and neural differentiation such as ASCL1 and NGN2 (Dhanesh et al., 2016), thus inhibition of Notch signaling is a necessary action for the generation of mature neurons.

A unique aspect of our transdifferentiation protocol is the presence of a progenitor stage characterized by neurosphere formation. Neurospheres are 3-D clusters of neural stem cells possessing the characteristics of self-renewal and multipotency (Bez et al., 2003). Since neurospheres were absent from cultures treated with small molecules alone we attribute the progenitor stage in our protocol to the action of ASCL1-IPTD. Furthermore, only neurons arising from neurospheres expressed mature neuronal markers MAP2 and NEUN by day 12. Passing through a progenitor stage has two advantages. First, the rapid metabolic transition that takes place during the fate switch from somatic cell to neuron puts enormous stress on the cell, leading to the formation of reactive oxygen species (ROS), known to induce toxicity and affect cell fate regulation, posing a major barrier to transdifferentiation. It stands to reason that an intermediate stage would reduce this oxidative stress, promoting a safer transition between cell fates and improving efficiency (Gascón et al., 2016). Second, the generation of neural stem cells improves the efficiency of the protocol since each neural stem cell can produce several neurons. *In vivo*, the effect of neural stem cells may also be to prolong the activity of neural regeneration, providing neurotrophic support and newly formed neurons for a sustained period of time (Abe, 2000; Lu et al., 2003; Chen et al., 2017).

Our transdifferentiation process was highly efficient in comparison with protocols using viral ASCL1 for similar applications. For instance, viral delivery of 3 transcription factors, ASCL1, BYRN2, and MYT1L, is reported to transdifferentiate MEFs into neurons after 22 days with an efficiency of 15–19% (Vierbuchen et al., 2010), while viral ASCL1 alone is reported to also transdifferentiate human fibroblasts into neurons by day 22, but at lower efficiencies (Chanda et al., 2014). Astrocytes have proven easier to transdifferentiate into neurons, and viral ASCL1 delivered to postnatal mouse astrocytes is reported to convert 77% into neurons by day 21 (Liu et al., 2015), and when delivered to human astrocytes (Sciencell #1800) in combination with the pluripotency transcription factor Nanog homeobox (NANOG) is reported to produce 41% neurons by day 15 (Corti et al., 2012). By comparison, our protocol generated 69% neurons from human astrocytes (Sciencell #1800) by day 12.

Small molecule protocols are reported to generate neurons from astrocytes with similarly high efficiencies, but with considerable cell death in the process, ultimately reducing total efficiency. For instance, Gao et al. used a combination of 6 small molecules (valproic acid, CHIR99021, repsox, forskolin, i-Bet151, and ISX9) to transdifferentiate human adult astrocytes into neurons in 12 days, reporting an efficiency of 70% of surviving cells, or 8% of the initial cell population (Gao et al., 2017). In contrast, Zhang et al. showed that by adding fewer small molecules in a sequential manner, cell death can be avoided in small molecule protocols. In their protocol they used a combination of 9 small molecules (LDN193189, SB431542, TTNPB, Thiazovivin, CHIR99021, DAPT, valproic acid, SAG and purmorphamine) in a step-wise manner to transdifferentiate human astrocytes (ScienCell #1800) into neurons after 14 days, with an efficiency of 67% (Zhang et al., 2015). They used the priming small molecules LDN193189 and SB431542 for days 1-2 similar to our protocol. They found by removing small molecules one at a time that the Notch signal inhibitor DAPT was the most significant enhancer of transdifferentiation, followed closely by the GSK3 signal inhibitor CHIR99021. GSK3 signaling represses canonical Wnt signaling and NGN2 expression, both key regulators of neurogenesis (Hirabayashi et al., 2004; Li et al., 2012; Valvezan and Klein, 2012), thus GSK3 inhibition can be used to promote neurogenesis. Surprisingly, our screening data showed that while either CHIR99021 or DAPT were necessary to enhance transdifferentiation with ASCL1-IPTD, when used together actually decreased transdifferentiation by ~50%. This suggests that although efficiencies are similar, the signaling pathways involved in our protocol differ substantially from the small molecule protocol.

Cooperation between ASCL1 and Notch signal inhibition can occur naturally in astrocytes *in vivo* after injury (Magnusson et al., 2014). Magnusson *et al*. identified Notch inhibition shortly after injury as a trigger for a latent neural transdifferentiaton program in resident astrocytes. The similarities between this latent program and the protocol we developed are striking. In their study, they observed a downregulation of Notch receptors and ligands in astrocytes followed by the appearance of ASCL1 expression after a stroke. These ASCL1-positive astrocytes clustered around the lesion and multiplied, giving rise to small, round DCX-positive neural stem cells by week 2, and eventually to mature NEUN-positive neurons by week 7, some of which expressed nitric oxide synthase (nNOS), a marker for GABAergic striatal interneurons. Based on these similarities, it is possible that our protocol takes advantage of this latent program in astrocytes by first recapitulating the plastic aspects of astrocytes at the site of an injury using SMAD inhibition, followed by the concerted action of ASCL1 upregulation by IPTD and Notch signal inhibition by DAPT. By this reasoning, it can be hypothesized that *in vivo* application of ASCL1-IPTD may be sufficient to trigger transdifferentiation of astrocytes at a site of injury into functional neurons. Although further work will be required to verify such a strategy, it would lend itself well to *in vivo* translation using a version of IPTD fitted with a domain to specifically target reactive astrocytes.

## Conclusion

Here we have shown a novel and non-viral protocol for efficient transdifferentiation of astrocytes into neurons. This protocol requires the delivery of only one transcription factor, ASCL1, by a novel PTD, and 4 small molecules, the SMAD inhibitors LDN193189 and SB431542, the Notch inhibitor DAPT, and the histone deacetylase inhibitor valproic acid. The efficiency of this protocol is enhanced significantly by the natural plasticity of astrocytes, suggesting its suitability for application *in vivo* where injuries lead to increased plasticity in reactive astrocytes. Future work will determine if the small molecules are necessary *in vivo*, and incorporate an astrocyte-targeting motif in the IPTD design. Further work will also adapt this protocol for producing specific neuronal subtypes through cooperation of ASCL1-IPTD with other IPTD-functionalized transcription factors.

## Author contributions

MR designed the experiments, analyzed results, and wrote the manuscript. IF assisted in background research, guidance on experimental design and analysis of the small molecule screening experiment. KS assisted in executing the ASCL-IPTD experiments on astrocytes. EM assisted in executing and analysing the NGN2-IPTD experiments on astrocytes. LC assisted in executing the ASCL1-IPTD experiments on MEFs and providing background research. SW provided experimental guidance, provided help writing and editing the final manuscript.

### Conflict of interest statement

This research was in part funded by an NSERC-CRD grant which we hold with iProgen Biotech who owns the PTD technology. This work was conducted in collaboration with iProgen Biotech who provided the functionalized transcription factors used in this work.

## Supplementary material

Supplemental Figure 1 Mouse embryonic fibroblasts (MEFs) express the skeletal myoblast gene myosin heavy chain 3 (MYH3) and lack the neuronal gene beta tubulin (TUJ1) after 14 days of exposure to ASCL1-IPTD and the cAMP activator forskolin, indicating that they have adopted a myogenic cell fate rather than a neuronal cell fate. **(A)** Exposure to 3 μg/mL ASCL1-IPTD is insufficient for transdifferentiation. Scale bar is 100μm. **(B)** Addition of forskolin results in transdifferentiation into myoblasts rather than neurons. Scale bar is 100μm. **(C)** Increasing the concentration of ASCL1-IPTD 5-fold still generates myoblasts rather than neurons. Scale bar is 100μm. **(D)** Increasing the concentration of ASCL1-IPTD 5-fold and adding forskolin still generates myoblasts rather than neurons. Scale bar is 100 μm **(E)** Control. Scale bar is 100 μm **(F)** Forskolin only control. Scale bar is 100 μm.
