# Supplementary figures and images for: Transdifferentiating Astrocytes Into Neurons Using ASCL1 Functionalized With a Novel Intracellular Protein Delivery Technology
Source: Front Bioeng Biotechnol. 2018 Nov 21;6:173. doi: 10.3389/fbioe.2018.00173 (PMC6258721; doi:10.3389/fbioe.2018.00173)

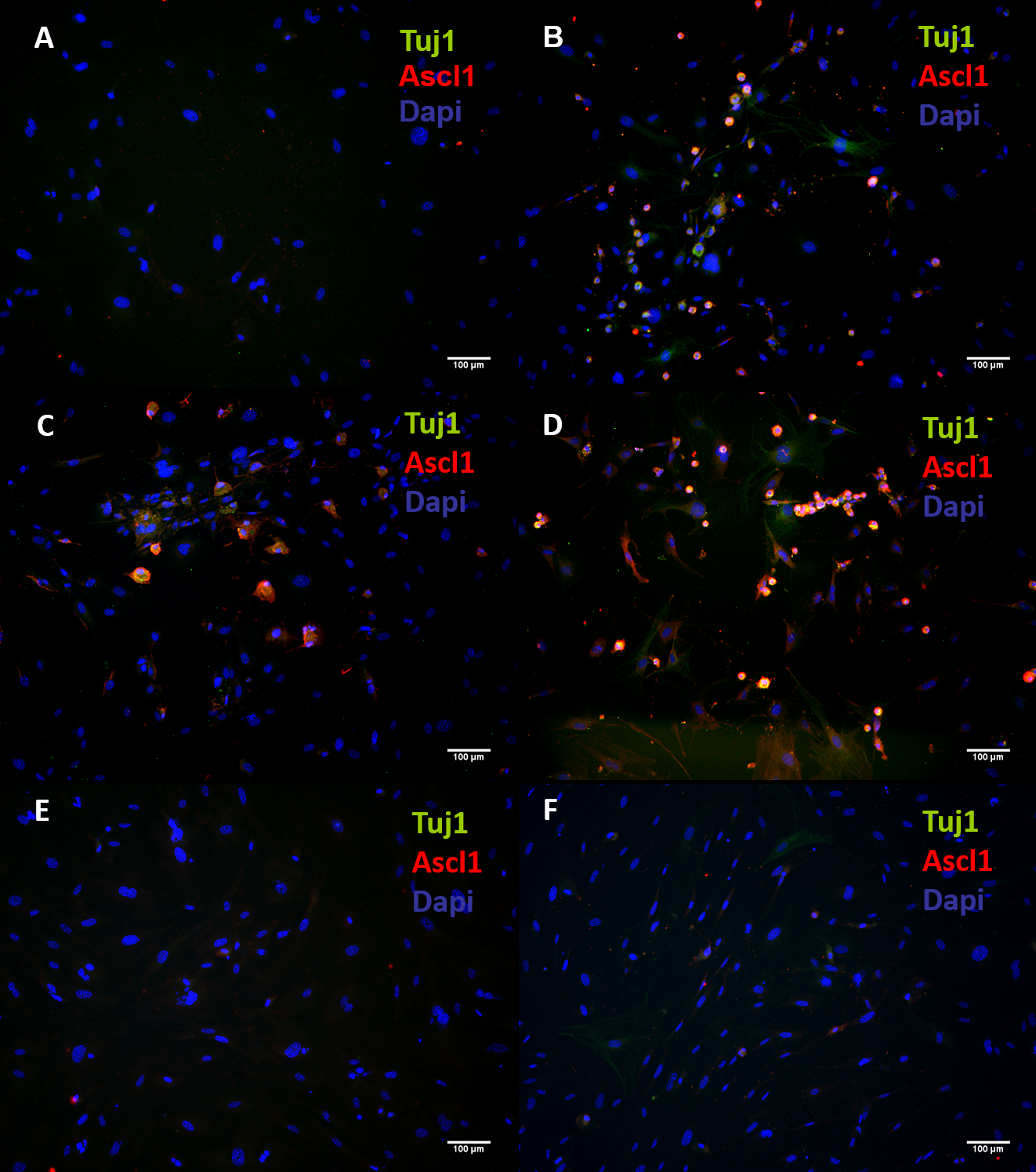

Supplement: Supplemental Figure 1 — Mouse embryonic fibroblasts (MEFs) express the skeletal myoblast gene myosin heavy chain 3 (MYH3) and lack the neuronal gene beta tubulin (TUJ1) after 14 days of exposure to ASCL1-IPTD and the cAMP activator forskolin, indicating that they have adopted a myogenic cell fate rather than a neuronal cell fate. (A) Exposure to 3 μg/mL ASCL1-IPTD is insufficient for transdifferentiation. Scale bar is 100μm. (B) Addition of forskolin results in transdifferentiation into myoblasts rather than neurons. Scale bar is 100μm. (C) Increasing the concentration of ASCL1-IPTD 5-fold still generates myoblasts rather than neurons. Scale bar is 100μm. (D) Increasing the concentration of ASCL1-IPTD 5-fold and adding forskolin still generates myoblasts rather than neurons. Scale bar is 100 μm (E) Control. Scale bar is 100 μm (F) Forskolin only control. Scale bar is 100 μm. [file Image_1.TIF]
